# Accidental Ingestion of Extended-Release Guanfacine in a Toddler

**DOI:** 10.7759/cureus.85261

**Published:** 2025-06-02

**Authors:** Dina R Assali, Alek Q Adkins, Jonathan Chang

**Affiliations:** 1 Emergency Department, OhioHealth Doctors Hospital, Columbus, USA; 2 Medical Toxicology Department, Nationwide Children’s Hospital, Columbus, USA; 3 Emergency Department, Nationwide Children’s Hospital, Columbus, USA

**Keywords:** bradycardia, guanfacine, hypertension, nicardipine, overdose

## Abstract

This case report describes the clinical presentation and treatment following an exploratory ingestion of extended release (ER) guanfacine in a healthy toddler. Guanfacine, an alpha-2a agonist, can cause transient hypertension as it moves from the serum into the CNS where it causes the more commonly seen central nervous system (CNS) depression, hypotension, and bradycardia. Persistent hypertension requiring intravenous (IV) vasodilatory medication is a rare and life-threatening complication of an alpha-2 agonist with only one other published report. This case report re-emphasizes and further expands available literature on this rarely reported effect.

## Introduction

Pediatric exploratory ingestions are among the more common toxicologic presentations in the emergency department (ED) [1]. It is often between the ages of two and five years that toddlers exhibit exploratory behaviors, namely, learning by eating, mostly unintentional and unaware of consequences. Many pharmacologic ingestions require pediatric intensive care unit (PICU) hospitalization for any abnormalities in hemodynamics, neurologic status, and/or respiratory concerns [1]. According to the Poison Control Center (PCC), in 2020, the top five most commonly ingested objects by children under five years of age included cosmetics/personal care products, household cleaning substances, analgesics, toys/foreign bodies/miscellaneous, and dietary supplements/herbals [2]. There are little to no real clinical trials involving toxicology of medication use in children, and some medications specifically do not have consistent side effects due to their mechanism of action. In this case report, we will focus on the tenuous nature of extended-release guanfacine in a presumed overdose of a toddler necessitating a nicardipine drip and PICU level of care. 

## Case presentation

**Figure 1:**
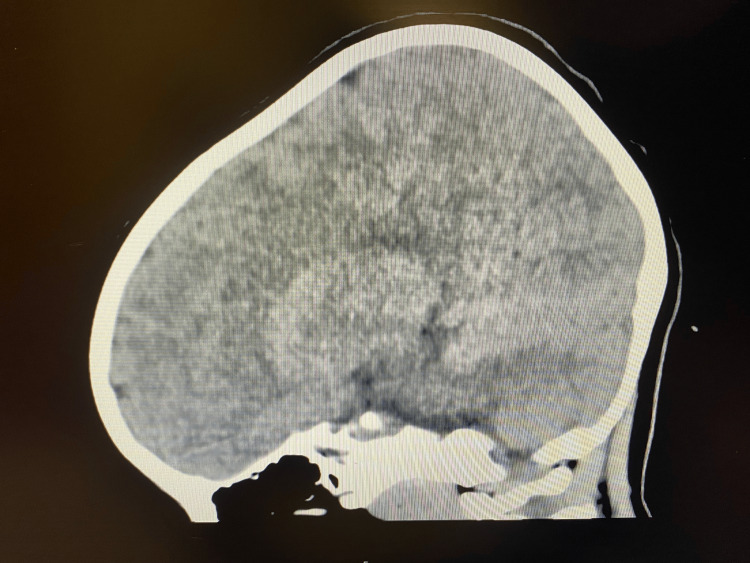
Non-contrasted computed tomography of the head in sagittal view

A three-year-old girl with no significant past medical history presented with lethargy, bradycardia, and hypertension to the ED. According to mom, she may have ingested approximately two to three tabs of 4 mg guanfacine ER two hours prior to arrival. The medication belonged to an older sibling and, per mom, was typically kept in the medicine cabinet. The mother was unsure how the patient was able to obtain the medication, but was able to confirm that there were several tabs missing after she administered the medication to the patient's sibling earlier in the day, while no other medications were missing. She called the local PCC, who advised that the patient be taken to the ED. On arrival to ED, the patient's initial vital signs included as follows: blood pressure 122/99 mmHg (reference: 89-112/46-72 mmHg), heart rate 64 beats per minute (reference: 80-120 beats per minute), respiratory rate about 16 per minute (reference: 20-28 per minute) with SpO2 of 92% (reference: 96% and above) on room air, and body temperature 97.6°F (reference: 98.6°-100.3°F). The child was minimally responsive to tactile stimuli on arrival but maintained a cough reflex and was protecting her airway. Due to concerns of co-ingestion given her depressed responsiveness and bradypnea, two doses of 0.2 mg IV naloxone were administered about five minutes apart without any improvement. Her initial workup, including complete blood count, comprehensive metabolic panel, lactic acid, venous blood gas, acetaminophen level, salicylate level, alcohol level, urinalysis, urine drug screen (Table 1), and a non-contrasted computed tomography of the head (Figure 1), was unremarkable.

**Table 1:** Urine drug screen results

Urine Drug Screen	Result
Amphetamines/methamphetamine	Not detected
Barbiturates	Not detected
Benzodiazepines	Not detected
Buprenorphine	Not detected
Cannabinoids	Not detected
Cocaine/metabolites	Not detected
Fentanyl	Not detected
Methadone	Not detected
Opiates	Not detected

During the workup, the patient developed worsening hypertension with systolic blood pressures >170 mmHg while in the ED. After discussion with the on-call medical toxicologist from the local PCC, a nicardipine drip was initiated at a starting rate of 0.5 mcg/kg/minute, and the patient was admitted to the PICU. As the patient’s blood pressure improved, her mental status improved, supporting our suspicion that the patient had hypertensive encephalopathy. After approximately 10 hours, her hemodynamics normalized, the nicardipine infusion was discontinued, and she was transitioned to a regular inpatient floor bed. She had returned to her normal self and tolerated food and drink. Repeat blood work was unremarkable for additional end-organ dysfunction from the patient’s hypertensive emergency. No further toxicologic workup was completed, given the patient's history reported by the mother, guanfacine was the likely cause of her symptoms. The patient was observed for an additional 24 hours, remained hemodynamically stable, and was discharged home on hospital day 2.

## Discussion

Guanfacine is a centrally and peripherally acting selective alpha-2a adrenergic agonist that was approved to treat attention-deficit/hyperactivity disorder (ADHD) in 2009 [3,4]. This medication was initially an anti-hypertensive with an off-label use for psychostimulation or for behavioral concerns in children [3]. In the US, there are immediate and extended release formulations with peak plasma concentrations from one to two hours to five hours, respectively. Centrally, guanfacine stimulates postsynaptic alpha-2a receptors in the prefrontal cortex, locus coeruleus, nucleus tractus solitarii, and the rostral ventrolateral medulla [3,5]. Guanfacine also acts as an imidazoline receptor agonist, hence its initial use as an anti-hypertensive medication [3]. This central alpha-2 and imidazoline agonism leads to decreased catecholamine release and sympathetic flow, which in turn causes hypotension and bradycardia [6]. Paradoxically, guanfacine also activates peripheral alpha receptors, leading to an increase in blood pressure via increased systemic vascular resistance. Hemodynamically speaking, this peripheral and central alpha receptor stimulation can produce a biphasic clinical picture of hypertension with reflex bradycardia followed by hypotension with bradycardia, as guanfacine moves from the serum compartment to the central nervous system.

Clinically, guanfacine overdose typically presents with drowsiness, lethargy, and diaphoresis [5]. Early in the presentation, there are case reports that have shown guanfacine to present with hypertension and reflex bradycardia [6-7]. It can then progress to hypotension and bradycardia seemingly as central nervous system levels rise [6-8]. This hypertension is usually transient and clinically insignificant, as mentioned in some case reports [6-9]. Although not common, this hypertension can persist and cause hypertensive encephalopathy. One proposed explanation of this persistent hypertension is continuous peripheral alpha receptor stimulation during the prolonged absorption phase of extended release formulations. Due to this biphasic nature, a typical observation period can vary. Overall, the mainstay of guanfacine overdose treatment is supportive care and monitoring for hemodynamic instability [4-10].

Previously, only one hypertensive crisis with concomitant hypertensive encephalopathy has been reported in an overdose [4], indicating that when present, hypertension can be severe. In this case report by Fein et al. [4], they used a nicardipine drip. Typically, an appropriate drug would include vasodilators or neuroprotective anti-hypertensives, such as nicardipine. In our case, we also used a nicardipine drip as we assumed the mother’s story to be true and that the patient’s mentation and hemodynamics were a result of the assumed guanfacine overdose. After being weaned from the nicardipine drip, she did not become hypotensive at any point, which is unlike other cases described previously [4,6-9]. This has also not been demonstrated before in an otherwise healthy toddler with a first-time exposure to the drug. One case of hypertensive encephalopathy in association with the medication was reported when withdrawing from guanfacine [11]. There are limitations to this case report. Firstly, we did not do any confirmatory drug testing aside from the aforementioned to verify that this patient truly took guanfacine ER or the number of tablets reported. Another limitation is that we assumed that the patient’s mentation improved directly from the resolution of her hypertension as a direct result of the nicardipine drip, when it could have also been attributed to the natural course of metabolism. Thirdly, this patient had an observation period of a little over 24 hours. The tenuous nature of this drug, through our understanding of the central and peripheral agonism seen in other case reports, serves as an example of the importance of extended hemodynamic and neurologic monitoring. This may be especially important for the extended-release formulation.

## Conclusions

Alpha-2 agonists - like guanfacine - have a paradoxical biphasic presentation. Providers must remain astute and be ready to treat both extremes of hemodynamic instabilities when evaluating patients after an ingestion, and a history that there are ADHD medications present in the home.
